# Microglia Dystrophy Following Binge-Like Alcohol Exposure in Adolescent and Adult Male Rats

**DOI:** 10.3389/fnana.2020.00052

**Published:** 2020-08-13

**Authors:** S. Alex Marshall, Justin A. McClain, Jessica I. Wooden, Kimberly Nixon

**Affiliations:** ^1^Department of Pharmaceutical Sciences, University of Kentucky, Lexington, KY, United States; ^2^Division of Pharmacology & Toxicology, College of Pharmacy, The University of Texas, Austin, TX, United States

**Keywords:** alcoholism, dystrophic, ethanol, hippocampus, microglia, neurodegeneration

## Abstract

Microglia are dynamic cells that have roles in neuronal plasticity as well as in recovery responses following neuronal injury. Although many hypothesize that hyperactivation of microglia contributes to alcohol-induced neuropathology, in other neurodegenerative conditions disruption of normal microglial processes also contributes to neuronal loss, particularly as microglia become dystrophic or dysfunctional. Based on the observation of a striking, abnormal morphology in microglia during binge-like ethanol exposure, the present study investigated the impact of excessive ethanol exposure on microglia number and dystrophic morphology in a model of alcohol dependence that includes neurodegeneration in both adult and adolescent rats. Following 2- and 4-day binge ethanol exposure, the number of microglia was decreased in the hippocampus and the perirhinal and entorhinal cortices of both adult and adolescent rats. Furthermore, a significant number of microglia with a dystrophic morphology were observed in ethanol-exposed tissue, accompanied by a significant decrease in brain-derived neurotrophic factor (BDNF) expression in the hippocampus. Together these findings suggest another means by which microglia may contribute to alcohol-induced neurodegeneration, specifically dystrophic microglia and/or loss of microglia may disrupt homeostatic and recovery mechanisms. These results demonstrate that microglia also degenerate with excessive alcohol exposure, which has important implications for understanding the role of microglia—and specifically their contributions to plasticity and neuronal survival—in neurodegenerative disease.

## Introduction

Excessive alcohol intake, a hallmark of alcohol use disorders (AUDs), produces neurodegeneration which may result in significant cognitive and behavioral impairments that contribute to the downward spiral from social alcohol drinking to addiction (Crews and Nixon, 2009). Neuroimmune activation is hypothesized to contribute to alcohol-induced neuropathology and therefore AUD development, especially in adolescents (Chastain and Sarkar, 2014; Crews et al., 2016). Adolescents often begin experimenting with alcohol despite their greater susceptibility to its damaging effects (Nixon and McClain, 2010; Crews et al., 2016), therefore determining the impact of alcohol on the neuroimmune system in adolescents vs. adults is critical to our understanding of how AUDs develop (Crews et al., 2016; Melbourne et al., 2019). The contribution of the neuroimmune system has been inferred from gene expression studies in human tissues and increased expression of microglial markers and chemokines in the brains of post-mortem alcoholics (e.g., Liu et al., 2004; He and Crews, 2008; Crews et al., 2013). In both adolescent and adult animal models of AUDs, microglia are activated by excessive ethanol consumption (McClain et al., 2011; Crews et al., 2013; Marshall et al., 2013; Peng et al., 2017; for review see Crews et al., 2016; Melbourne et al., 2019) and manipulating neuroimmune signaling drives alcohol consumption in some models (Agrawal et al., 2011; Blednov et al., 2011). The specific role of microglia, however, has been less clear (Walter and Crews, 2017; Warden et al., 2020).

Microglia are dynamic cells best known for their role as initial responders to insult in the central nervous system (CNS; Tremblay et al., 2011; Li and Barres, 2018). Their extended processes survey and influence the surrounding microenvironment and communicate directly with neuronal cell bodies (Nimmerjahn et al., 2005; Cserép et al., 2020). Recent discoveries describe their critical role in plasticity, including remodeling and pruning synapses, mediating neurotransmission, and influencing adult neurogenesis (Tremblay et al., 2011; Zhan et al., 2014; Li and Barres, 2018; Cserép et al., 2020). As such, microglia make important contributions to brain function such that microglial dysfunction would have detrimental effects on neuronal plasticity and associated brain functions. Although activation of these cells and their roles in secondary injury cascades are the focus of most reports, disruption of microglia or their processes can be detrimental to repair/recovery, resulting in worse outcomes (Tremblay et al., 2011). Indeed, deterioration of microglial function with aging may be a contributing factor in neurodegenerative diseases such as Alzheimer’s disease (Streit and Xue, 2009). These dystrophic microglia are hypothesized to have impaired phagocytic and proinflammatory responses but also a reduced capacity to release anti-inflammatory or neurotrophic factors vital for cellular repair (Streit and Xue, 2009). As acute microglial activation has been described as necessary for “housekeeping” in the CNS (Tremblay et al., 2011), the lack of phagocytic activity following alcohol-induced brain damage could be indicative of microglial dysfunction.

While examining microglia morphology in work that determined the effect of binge-like alcohol exposure on microglia activation state (McClain et al., 2011; Marshall et al., 2013), many cells with an unexpected morphology were observed, an effect consistent with microglia dystrophy. Dystrophy is associated with microglial cells that exhibit cytorrhexis and/or a variety of morphologic aberrations, including processes that are de-ramified, fragmented, beaded, or stripped down (Streit and Xue, 2009). In this previous work (McClain et al., 2011; Marshall et al., 2013), we noted microglia with cytoplasmic extensions that form spherical swellings, or “beaded” processes, which indicate a partial fragmentation of the processes (Streit et al., 2004). Therefore, we examined the effect of binge-like ethanol exposure on microglia numbers in the hippocampus and perirhinal and entorhinal (peri-entorhinal) cortices of adolescent and adult rats. These regions were selected as previous research reports alcohol-induced neurodegeneration in these regions in this model (Obernier et al., 2002; Hayes et al., 2013; West et al., 2019).

## Materials and Methods

### Binge Alcohol Model

Male, Sprague–Dawley adult (approximately postnatal day 70; *n* = 44) or adolescent (postnatal day 35; *n* = 31; Charles River Laboratories, Raleigh, NC, USA) rats were subjected to the Majchrowicz AUD model as reported (Morris et al., 2010; Hayes et al., 2013). Briefly, rats were gavaged with ethanol (25% w/v in Vanilla Ensure Plus^®^, Abbott Laboratories, Abbott Park, IL, USA) or isocaloric control diet every 8 h for 2 days or 4 days. Following their initial 5 g/kg ethanol dose, subsequent doses were titrated according to a 6-point intoxication behavior scale. Blood ethanol concentrations (BECs) were determined in serum (AM1 Alcohol Analyser; Analox, London, UK), from tail blood collected 90 m following the seventh dose of ethanol (4 days) or from trunk blood (2 days). All experiments were approved by the University of Kentucky Institutional Animal Care and Use Committee.

### Immunohistochemistry

Rats were overdosed with sodium pentobarbital (Fatal Plus^®^, Vortech Pharmaceuticals, Dearborn, MI, USA; i.p.) and transcardially perfused with phosphate-buffered saline then 4% paraformaldehyde in 0.1 M phosphate buffer (pH 7.4) immediately following the last dose of ethanol. Tissue harvesting and immunohistochemistry on 40 μm free-floating coronal sections followed published methods (McClain et al., 2011; Marshall et al., 2013). Every twelfth section, such that sections were 480 μm apart, underwent diaminobenzidine-labeled (Polysciences, Waltham, MA, USA) immunohistochemistry for Iba1 (1:1,000; Wako, Richmond, VA, USA), a calcium-binding protein found in all microglia. An adjacent series of adult rat tissue was processed for OX42 (Complement Receptor 3; 1:1,000; BioRad, Hercules, VA, USA) immunoreactivity and reported previously (Marshall et al., 2013).

The number of Iba1-positive (Iba1+) cells was estimated using Image-Pro Plus 6.3 (Media Cybernetics, Rockville, MD, USA) as previously reported (Marshall et al., 2013). Identical percent change is found using this method vs. the optical fractionator method when sections are obtained in an unbiased manner (Crews et al., 2004; Marshall et al., 2013). Briefly, multi-panel images were obtained at 100× (Visiopharm, Hoersholm, Denmark) of the peri-entorhinal cortex (all sections from Bregma −3.00 to −6.00; 3–5 sections/brain) and dorsal hippocampus (all sections from approximately Bregma −2.52 to −4.56; 4–7 sections/brain). In each section, hippocampal cornu ammonis fields (CA1 and CA2/3), dentate gyrus, and peri-entorhinal cortex were circumscribed and the number of Iba1+ cells in each region was determined (cells/section).

During quantification, Iba1+ cells with beaded processes were noted in the hippocampus only. Therefore, the number of these dystrophic profiles was counted in the dorsal hippocampus of both adults and adolescents using a 60× oil immersion lens. Profile counts were obtained due to the low number and non-homogeneous distribution of these cells and reported as the number of dystrophic cells/section (Noori and Fornal, 2011). Slices previously analyzed and reported for OX42 densitometry (Marshall et al., 2013) were qualitatively assessed to ensure that dystrophic cells were not unique to the Iba1 construct.

### Enzyme-Linked Immunosorbent Assay

Hippocampi of a four-day binge, adult rats were dissected, snap-frozen, and homogenized as previously reported (McClain et al., 2011; Marshall et al., 2013). Homogenates were centrifuged at 20,000× *g* for 15 mins at 4°C, and the supernatant stored at −80°C. Total protein levels were determined using a Pierce BCA Protein Assay Kit (Thermo Scientific, Rockford, IL, USA), while brain-derived neurotrophic factor (BDNF) content was determined *via* ELISA (#CYT306 Millipore, Billerica, MA, USA) following the manufacturer’s instructions, normalized to total protein determined by BCA Assay, and expressed as pg of BDNF/mg of protein.

### Statistical Analysis

Prism (V.5.04, GraphPad Software, Inc., LaJolla, CA, USA) was used for all analyses. Intoxication behavior data were non-parametric and therefore analyzed by the Kruskal–Wallis H test (ANOVA for ranks) followed by Dunn’s *post hoc* test. BECs, cell counts, and morphology were analyzed with two-way ANOVAs (with the variables of time and treatment) followed by *post hoc* Bonferroni tests. BDNF levels were compared using a *t*-test. All data were expressed as mean ± SEM and significance accepted at *p* < 0.05.

## Results

### Animal Model Data

As adolescents are less sensitive than adults to sedative and motor-impairing effects of ethanol, the Majchrowicz model was used as it overcomes these differences and results in similar BECs (Morris et al., 2010). Thus, as expected, adolescent rats differed from adults in intoxication behavior (H = 22.65, *p* < 0.0001), where Dunn’s *post hoc* test revealed a lower intoxication score for adolescents (i.e., less sedative/motor impairment). As the dose is inversely related to intoxication score, the mean daily ethanol dose was lower in adults vs. adolescents as indicated by a main effect of age (*F*_(1,34)_ = 46.01, *p* < 0.0001); however, no main effect for the duration of exposure (i.e., 2 or 4 days) or age × duration interaction was observed. For BECs, 4 days of exposure produced higher BECs than 2 days according to the main effect of duration (*F*_(1,34)_ = 6.99, *p* = 0.01), but no main effect of age or age × duration interaction was found (Table 1).

**Table 1:** Animal model parameters.

	2 Days		4 Days	
Parameter	Adults (*n* = 8)	Adolescents (*n* = 8)	Adults (*n* = 14)^1^	Adolescents (*n* = 8)
Intoxication behavior*	2.0 ± 0.2	1.3 ± 0.2	2.0 ± 0.2	1.0 ± 0.1
Dose (g/kg/day)*	9.8 ± 0.5	11.8 ± 0.6	9.1 ± 0.4	12.2 ± 0.2
BECs (mg/dL)^#^	299.5 ± 18.6	302.7 ± 31.2	389.5 ± 17.2	329.5 ± 19.2

^1^Collapsed across ELISA (n = 7) and IHC (n = 7) studies. *Main effect of age; ^#^main effect of exposure duration, p < 0.05.

### Ethanol Decreases the Number of Microglia

**Figure 1:**
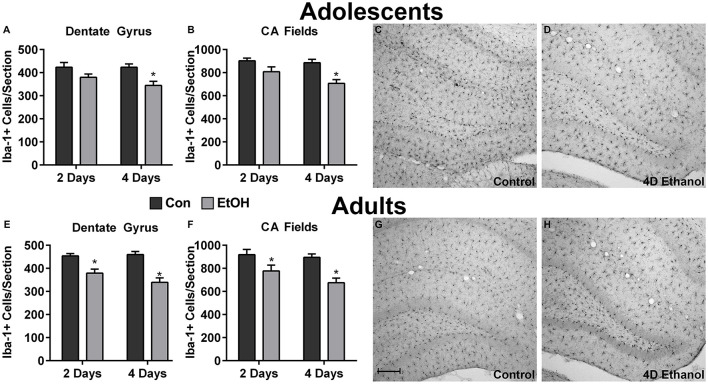
Ethanol decreases microglia numbers in the hippocampus. Ethanol-exposed rats (light gray bars) had fewer Iba1+ cells compared to controls (dark bars) in both adolescents **(A,B)** and adults **(E,F)**. *Post hoc* tests indicated that Iba1+ cells were reduced after 4 days in the adolescent dentate gyrus **(A)** and CA fields **(B)** and after both the 2 and 4-day binge in adults in both regions **(E,F)**. Representative photomicrographs of the dentate gyrus of control-treated adolescent **(C)** and adult **(G)** animals have significantly more microglia compared with the photomicrographs of the alcohol-treated corresponding adolescent **(D)** or adult **(H)** images. Data are mean ± SEM. Scale bar = 200 μm, *p < 0.05.

**Figure 2:**
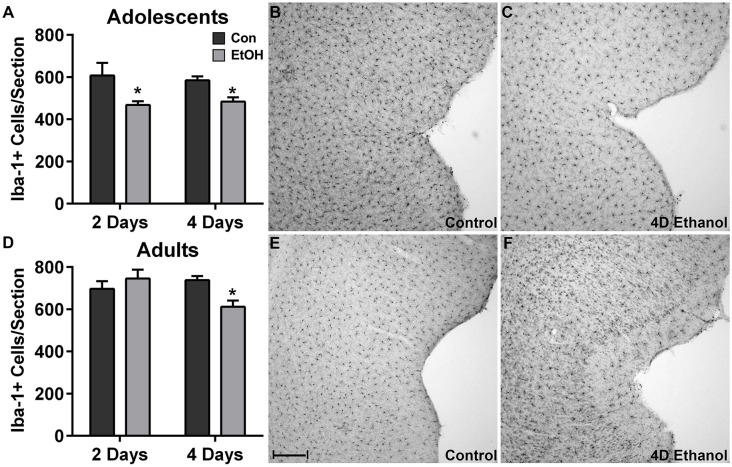
Ethanol decreases microglia numbers in the peri-entorhinal cortex. In the peri-entorhinal cortex, *post hoc* tests indicated a reduced number of microglia after both 2 and 4 days of ethanol exposure vs. controls in adolescents **(A)** while the loss of microglia occurred after only 4-days of ethanol exposure in adults **(D)**. Representative photomicrographs of the entorhinal cortex of adolescent **(B)** and adult **(E)** control-treated animals have significantly more microglia compared with the photomicrographs of the alcohol-treated corresponding adolescent **(C)** or adult **(F)** images. Data are mean ± SEM. Scale bar = 200 μm, *p < 0.05.

To investigate the effect of ethanol on the number of microglia in the hippocampus and peri-entorhinal cortex, Iba1+ cells were counted. Qualitatively and as reported previously, microglia in ethanol-treated rats appeared activated but not to an M1-like, amoeboid state: Iba1+ cells appear more compact, though maintain a ramified morphology (McClain et al., 2011; Tremblay et al., 2011; Marshall et al., 2013). However, the number of Iba1+ cells was decreased by approximately 20% in adolescents and adults across multiple subregions of the hippocampus in ethanol-treated animals (Figure 1). Two-way ANOVAs indicated a main effect of treatment (i.e., ethanol exposure) on Iba1+ cell number in the dentate gyrus of adolescents (*F*_(1,27)_ = 13.78, *p* = 0.0009) and adults (*F*_(1,26)_ = 40.38, *p* < 0.0001) and in CA fields of adolescents (*F*_(1,27)_ = 16.79, *p* = 0.0003) and adults (*F*_(1,26)_ = 13.78, *p* < 0.001), but no main effects of duration of exposure nor an interaction (treatment × duration). *Post hoc* tests revealed that this decrease was significant after only 4 day ethanol exposure in adolescents (Figures 1A,B) but occurred after 2 days and 4 days in adults (Figures 1E,F). In the peri-entorhinal cortices (Figure 2), a significant main effect of treatment was observed in adolescents (*F*_(1,27)_ = 16.79, *p* = 0.0003) and adults (*F*_(1,26)_ = 13.78, *p* < 0.001), but no main effect of duration nor interactions (treatment × time) were observed. *Post hoc* tests revealed Iba1+ cells were reduced after 2 day and 4 day ethanol exposure in adolescents (Figure 2A); whereas Iba1+ cells were decreased after only 4 day ethanol exposure in adults (Figure 2D).

### Increase in Dystrophic Microglia With Ethanol Exposure

**Figure 3:**
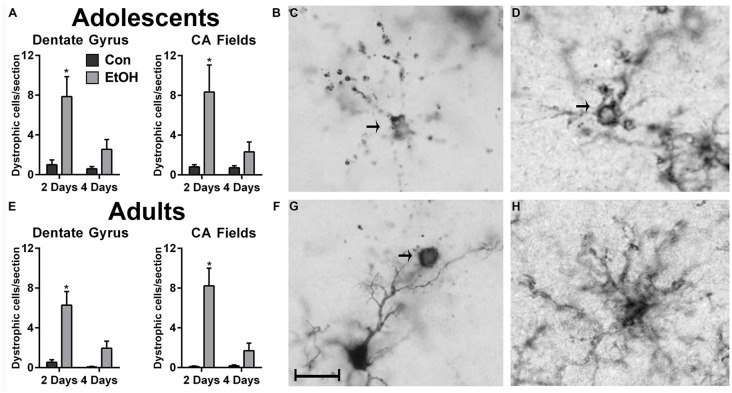
Ethanol increases the number of dystrophic microglia. Rats exposed to ethanol had more Iba1+ dystrophic microglia in both adolescents **(A,B)** and adults **(E,F)** compared with the controls. Arrows in photomicrographs indicate Iba1+ dystrophic microglia as signified by beaded processes and/or cytorrhexis in panels **(C,D,G)**. Although dystrophic cell counts were based on Iba1+ cells, a secondary microglial marker, OX42, also showed the presence of cytorrhexis in 4-day binge ethanol exposed adult rats **(D)** compared with a normal ramified microglial cell in adult controls **(H)**. Data are mean ± SEM. Scale bar = 20 μm, *p < 0.05.

In the hippocampus, microglia with a cytorrhexic or beaded morphology were counted as dystrophic (Streit and Xue, 2009) and were observed in all treatment groups (Figure 3). An increase in the number of dystrophic microglia was observed following 2d of ethanol exposure among adolescents (Figures 3A,B) and adults (Figures 3E,F). For the number of dystrophic microglia, two-way ANOVAs indicated main effects of both time and treatment for adult (Time: *F*_(1,26)_ = 8.71, *p* = 0.007; Treatment: *F*_(1,26)_ = 21.99, *p* < 0.0001) and adolescents (Time: *F*_(1,27)_ = 5.87, *p* = 0.02; Treatment: *F*_(1,27)_ = 13.87, *p* < 0.001) in the dentate gyrus, as well as a treatment × duration interaction for adults (*F*_(1,26)_ = 5.65, *p* = 0.03) and adolescents (*F*_(1,27)_ = 4.29, *p* = 0.048) in the dentate gyrus. In the CA fields, main effects of treatment (*F*_(1,26)_ = 22.16, *p* < 0.0001) and duration (*F*_(1,26)_ = 9.94, *p* = 0.004) were revealed in adults but not adolescents, which only had a main effect of treatment (*F*_(1,27)_ = 9.21, *p* = 0.0005). Also in the CA fields, a treatment × time interaction was only observed in adults (*F*_(1,26)_ = 10.39, *p* = 0.03). Importantly, dystrophic morphology was not unique to the calcium-binding antibody, Iba1 (Figures 3C,G), as OX42+ microglia also showed evidence of cellular fragmentation (Figure 3D).

### BDNF Decreased by Ethanol Exposure

The impact of microglia dystrophy and loss was assessed by investigating BDNF protein expression *via* ELISA after 4d ethanol exposure in adult rat hippocampi. A 25% decrease in BDNF expression was observed between controls (264 ± 8.2 pg BDNF/mg protein; *n* = 5) and ethanol exposed rats (199 ± 11.5 pg BDNF/mg protein; *n* = 7; *t*_(10)_ = 4.22, *p* = 0.0018).

## Discussion

We report that excessive alcohol exposure results in dystrophic microglia in the hippocampus, an effect that was coupled with a loss of Iba1+ cells in the hippocampus as well as the peri-entorhinal cortex of adolescent and adult rats. This reduction in Iba1+ cells combined with the observation of a dystrophic morphology with two independent markers of microglia (Figure 3) supports that a loss of microglia occurs with binge-like alcohol exposure, which aligns with previous work in various models (Marshall et al., 2016; Barton et al., 2017; Grifasi et al., 2019; Hu et al., 2020). Although dystrophic microglia were not observed in the peri-entorhinal cortex, they may be present at a different time point considering the similar decrease in microglia number. In the 4-day binge, this loss occurs despite the remaining microglia showing evidence of activation, specifically increased OX42 immunoreactivity and [^3^H]PK-11195 binding to the translocator protein 18 (Marshall et al., 2013, 2016) similar to other neurotoxins (Verina et al., 2011). This activation is apparent in Figures 1 and 2 where the remaining Iba1+ cells appear more compact but still ramified in the ethanol-exposed groups, consistent with morphological changes that occur with activation, namely thickened processes and enlargement of the cell body, though immunoreactivity was not measured in this study. Loss or dysfunction of microglia has only recently been considered for a role in alcoholic neuropathology (Marshall et al., 2016; Barton et al., 2017; Grifasi et al., 2019; Hu et al., 2020), though others have long since proposed that deterioration of microglial function contributes to neurodegenerative disease (Streit and Xue, 2009; Butovsky and Weiner, 2018; Li and Barres, 2018). Thus, the loss of microglia observed here may contribute to the diminished microglial response to alcohol insult. Additionally, microglial dysfunction resulting from activation impacts both synaptic strength and neuronal excitability (Baalman et al., 2015; Clark et al., 2015), which further highlights the potential for glial cells to contribute to neurodegenerative states. Although a hyperactive neuroimmune response has been proposed as a potential source of alcohol-induced neurodegeneration, this report, when considered with our more extensive investigation of microglia activation (McClain et al., 2011; Marshall et al., 2013, 2016; Peng et al., 2017), argues that depression of the microglial response may also play a role in alcohol-induced neurodegeneration as well as the development of AUDs.

Following an insult such as the cell death observed in the hippocampus and peri-entorhinal cortex in the AUD model used (Obernier et al., 2002), microglia are expected to “react,” that is, migrate to these regions, remove cellular debris, and provide trophic support to salvageable cells (Streit and Xue, 2009; Tremblay et al., 2011; Li and Barres, 2018). Indeed, in this model, microglia hone to these sites of damage as well as other cortical regions and show a trophic phenotype during abstinence/recovery (Nixon et al., 2008; McClain et al., 2011; Marshall et al., 2013, 2016). The observation of dystrophic cells coupled with a reduced number of microglia, overall, indicates that not only are microglia not surmounting the expected response but that ethanol may be directly cytotoxic to microglia. Thus, during alcohol intoxication, microglia capacity to provide support may be limited. This idea is supported further by a lack of phagocytic microglia after the 4-day binge (Marshall et al., 2013, 2016) and the well-established alcohol-induced impairment in phagocytosis in the peripheral immune system (Goral et al., 2008).

As a means of assessing the potential impact of a loss of microglia, BDNF was measured in the hippocampus where dystrophic microglia were observed. Although microglia activation typically upregulates neurotrophins, including BDNF, following injury (Streit and Xue, 2009; Tremblay et al., 2011), we observed a 25% decrease in BDNF protein at this specific time point (see also a 15% but non-significant decrease in BDNF protein from adolescent binge hippocampus at 2 days post 4-day binge in McClain et al., 2014). These findings concur with some AUD models showing decreased BDNF in the CNS as well as the decreased BDNF in the serum of human alcoholics (Davis, 2008). BDNF is also secreted by other cell types that are dysregulated in this AUD model (Crews and Nixon, 2009; Geil et al., 2014), therefore the decrease in BDNF cannot be attributed solely to lost or damaged microglia. The loss of hippocampal microglia and/or BDNF could significantly impact hippocampal structure or function. In this AUD model, hippocampal neural stem cell proliferation and newborn neurons, survival is reduced in both adolescents and adults (Geil et al., 2014), which may be attributable to microglia and/or BDNF’s critical role in adult neurogenesis (Somkuwar et al., 2016). The inhibition of adult neurogenesis through decreased trophic support is just one example of how dysfunction of microglia may contribute to alcohol-induced neurodegeneration.

Experimentation with drugs of abuse, such as alcohol, often originates in adolescence; therefore, examining the adolescent and adult neurobiological responses to excessive ethanol exposure are critical to understanding how AUDs develop. Previous reports show that this AUD model causes similar low-grade or non-classical microglial activation in both adolescents (McClain et al., 2011; Peng and Nixon, unpublished observations) and adults (Marshall et al., 2013; Peng et al., 2017) that persists for weeks in abstinence. These data support that short-term exposure (2 days) to high BECs decreases the number of microglia in the hippocampus and peri-entorhinal cortex, an effect that was only previously observed after over weeks or 2 months of binge-like exposure in adolescent rats (Teixeira et al., 2014; Hu et al., 2020). These short-term effects in adolescents are particularly disturbing considering the persistence of developmental microglial effects on the adult brain and behavior (Bilbo and Schwarz, 2009). Whether alcohol-induced microglia loss, dysfunction, or the results of the combination of the two persist in the long term is not known. Signs of microgliosis, as well as effects on neuronal survival and astrogliosis, vary across different points of abstinence as well as with different patterns of exposure (Kelso et al., 2011; Marshall et al., 2013; Melbourne et al., 2019), suggesting that future studies should consider the transient or permanent nature of microglial loss and dystrophic cells caused by ethanol. Furthermore, the complex dynamics between activated microglia and microglial dysfunction are unknown. These data, however, suggest a new view on how microglia may be involved with alcohol-induced neurodegeneration as well as altered neuroplasticity (Tremblay et al., 2011; Zhan et al., 2014). Specifically, we postulate that during intoxication, alcohol-diminished microglial responses may exacerbate degeneration through the loss of trophic support. Therefore, microglia appear to play multiple roles in AUDs and associated neurodegeneration.

## Data Availability Statement

The raw data supporting the conclusions of this article will be made available by the authors, without undue reservation.

## Ethics Statement

The animal study was reviewed and approved by the University of Kentucky Institutional Animal Care and Use Committee.

## Author Contributions

SM, JM, and KN conceived and designed the experiments. SM and JM performed the experiments and analyzed the data. SM, JM, JW, and KN contributed to the interpretation of the data, writing, and editing the manuscript.

## Conflict of Interest

The authors declare that the research was conducted in the absence of any commercial or financial relationships that could be construed as a potential conflict of interest.
